# Three complete plastome sequences from the families of Lamiaceae, Mazaceae, and Phrymaceae (Lamiales)

**DOI:** 10.1080/23802359.2020.1861563

**Published:** 2021-01-19

**Authors:** Sangjin Jo, Hoe-Won Kim, Young-Kee Kim, Se-Hwan Cheon, Min-Jung Joo, Ja-Ram Hong, Myounghai Kwak, Ki-Joong Kim

**Affiliations:** a Division of Life Sciences, Korea University, Seoul, South Korea; b Department of Plant Resources, National Institute of Biological Resources, Incheon, South Korea

**Keywords:** Plastome, Lamiales, *Vitex rotundifolia*, *Phryma leptostachya* subsp. *asiatica*, *Mazus pumilus*

## Abstract

In this study, we report the first complete plastome sequence of *Vitex rotundifolia* (Lamiaceae) (MT937186). In addition, the plastome sequences of *Phryma leptostachya* subsp. *asiatica* (Phrymaceae) (153,324 bp; MT948145) and *Mazus pumilus* (Mazaceae) (152,847 bp; MT937187) are also included. The gene orders and structures of the three plastomes are collinear with those of the typical plastome of angiosperm. The plastome size of *V. rotundifolia* is 154,370 bp in length and consists of a large single-copy region of 85,079 bp and a small single-copy region of 17,917 bp, which are separated by a pair of 25,687 bp-long inverted repeat regions. In addition, the plastome sizes of *P. leptostachya* subsp. *asiatica* and *M. pumilus* are 153,324 bp and 152,847 bp, respectively. The three plastomes contain 113 genes, including 79 protein-coding, 30 tRNA, and four rRNA genes. Sixteen genes contain one intron and two genes have two introns. A total of 41 simple sequence repeat loci was identified in the *V. rotundifolia* plastome. Phylogenetic analysis shows that Viticoideae is a sister group of the last of Lamiaceae except Nepetoideae. The Mazaceae are a sister group of Lamiaceae, while Phrymaceae form a sister group to the Paulowniaceae-Orobanchaceae clade.

*Vitex rotundifolia* L.f. is a small shrub and native to seashores throughout the pacific side of Northeast Asia. It belongs to the family Lamiaceae in the order Lamiales (APG IV 2016). Lamiaceae consist of 12 subfamilies, 241 genera, and approximately 7530 species (Christenhusz and Byng 2016). The genus *Vitex* belongs to the subfamily Viticoideae of Lamiaceae. *Phryma leptostachya* L. subsp. *asiatica* (H. Hara) Kitamura (Phrymaceae) is a medium size annual herb and native to Northeastern America and Asia. It belongs to the family Phrymaceae in the order Lamiales (APG IV 2016). *Mazus pumilus* (Burm.f.) Steenis (Mazaceae) is a small annual herb in the open damp locations and native to South and East Asia. It belongs to the family Mazaceae in the order Lamiales (APG IV 2016). *Phryma leptostachya* subsp. *asiatica* (Phrymaceae) and *Mazus pumilus* (Mazaceae) belong to the sister families of Lamiaceae.

Samples of *V. rotundifolia*, *P. leptostachya* subsp. *asiatica*, and *M. pumilus* were collected in South Korea and their GPS co-ordinations are N33°18′12.6″-E126°48′33.6″, N35°16′35.7″-E127°28′35.0″, and N37°19′18.5″-E127°15′44.6″, respectively. The fresh leaves were ground into powder in liquid nitrogen and total DNAs were extracted using the G-spin^TM^ IIp for Plant Genomic DNA Extraction Kit (iNtRON Biotechnology, Seongnam-si, South Korea). The voucher specimens were deposited in the Korea University Herbarium (KUS acc. nos. 2008-0463, 2008-0813, and 2008-1240) and their genomic DNAs were deposited in the Plant DNA Bank in Korea (PDBK acc. no. 2008-0463, 2008-0813, and 2008-1240). NGS sequencings were performed using an Illumina MiSeq platform (Illumina Inc., San Diego, CA). Approximately, 100 ng of extracted DNA were used for library construction and raw sequence reads were generated using Illumina MiSeq using reagent kit v3 (600-cycles) (Illumina, Inc., San Diego, CA). The average length of raw reads is 301 bp. De novo assemblies and annotations of plastome were performed using the Geneious 11.1.5 (Biomatters Ltd., Auckland, New Zealand; Kearse et al. 2012), National Center for Biotechnology Information (NCBI) BLAST, and tRNAscan-SE programs (Lowe and Eddy 1997). The plastome of *Sesamum indicum* (NC016433) was used as a reference genome for annotation (Yi and Kim 2012). The average plastome coverage of *V. rotundifolia, P. leptostachya* subsp. *asiatica.*, and *M. pumilus* is 2003×, 505×, and 778×, respectively. The simple sequence repeats (SSRs) were detected with the Phobos v. 3.3.12 program (Leese et al. 2008) in the Geneious 11.1.5. For the phylogenetic analysis, we selected and downloaded 27 related complete plastome sequences based on the APG IV system (APG IV 2016) from the NCBI database.

The gene orders and structures of the three plastomes are collinear to those of typical angiosperms (Shinozaki et al. 1986; Kim and Lee 2004; Yi and Kim 2012). The complete plastome of *V. rotundifolia* is 154,370 bp in length, and consists of a large single-copy (LSC) region of 85,079 bp and a small single-copy (SSC) region of 17,917 bp, which are separated by two inverted repeats (IRs) of 25,687 bp. The plastome comprises 113 unique genes (79 protein-coding genes, 30 tRNA genes, and four rRNA genes). Six protein-coding, seven tRNA, and four rRNA genes are duplicated in the IR regions. The average A-T content of the plastome is 61.7%, whereas that in the LSC, SSC, and IR regions is 63.6%, 67.3%, and 56.7%, respectively. Sixteen genes contain one intron and two genes, *ycf3* and *clpP*, have two introns. A total of 41 SSR loci are distributed throughout the plastome. Among these, 35, 5, and 1 are mono-SSR, di-SSR, and tri-SSR loci, respectively.

The complete plastomes of *P. leptostachya* subsp. *asiatica* (153,324 bp; MT948145) and *M. pumilus* (152,847 bp; MT937187) were also fully assembled and annotated. The plastome size of *P. leptostachya* subsp. *asiatica* is 157 bp longer than the previously reported same species (Xia et al. 2019). In contrast, the plastome size of *M. pumilus* was 187 bp shorter than the previously reported same species (Xia et al. 2019). These differences probably reflect the geographic differences between Korean and Chinese populations. To validate the phylogenetic relationships of three species, 30 whole plastome sequences including all noncoding regions were aligned as a single data matrix (178,589 bp in length) using the MAFFT v. 7.017 in Geneious v. 11.1.5 (Biomatters Ltd., Auckland, New Zealand; Kearse 2012). Then, a maximum-likelihood (ML) tree was reconstructed by RAxML v.8.2.12 in CIPRES webserver (Stamatakis 2014) using the GTR + G + I model with 1000 bootstrap replicates. The resulting tree shows that *V. rotundifolia* (Viticoideae) is a sister group of the last of Lamiaceae except Nepetoideae (Figure 1). In previous studies, Viticoideae show a close relationship to Symphorematoideae (Li et al. 2016; Zhao et al. 2020). Mazaceae (*M. pumilus*) were the sister family of Lamiaceae, while Phrymaceae (*P. leptostachya* subsp. *asiatica*) form a sister group to the Paulowniaceae-Orobanchaceae clade. The complete plastome sequence in this report will provide a useful resource for the phylogenetic and evolutionary studies of Lamiales.

**Figure 1.**
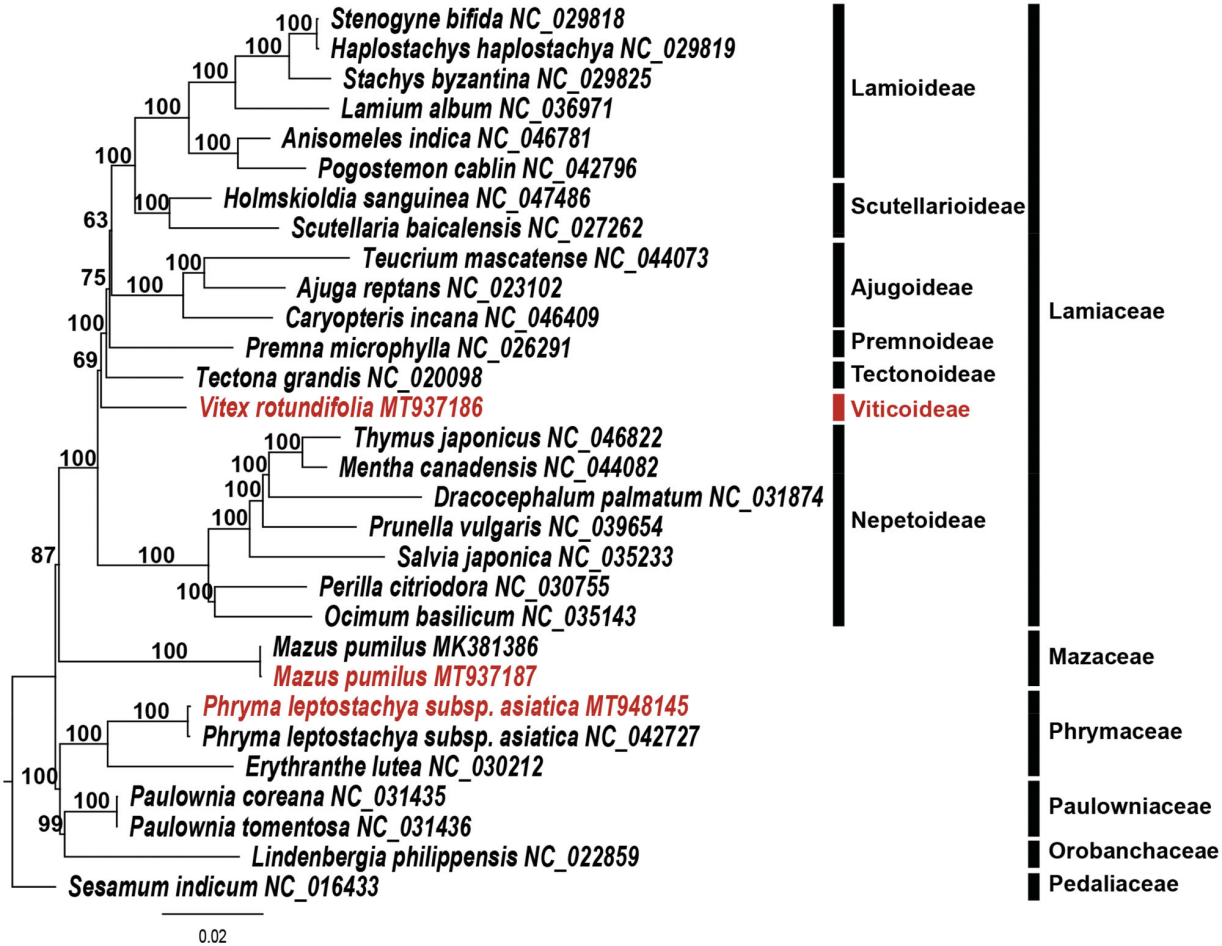
Maximum-likelihood (ML) tree based on 30 whole plastome sequences of Lamiales as determined by RAxML(–ln *L =* 700772.904266). The numbers at each node indicate the ML bootstrap values.

## Data Availability

The data that support the finding of this study are openly available in GenBank of NCBI at https://www.ncbi.nlm.nih.gov, reference number MT937186 for *Vitex rotundifolia*, MT948145 for *Phryma leptostachya* subsp. *asiatica*, and MT937187 for *Mazus pumilus*.
